# Supplementary material for: The causal role of circulating inflammatory markers in osteoporosis: a bidirectional Mendelian randomized study
Source: Front Immunol. 2024 Jul 18;15:1412298. doi: 10.3389/fimmu.2024.1412298 (PMC11291241; doi:10.3389/fimmu.2024.1412298)
Supplement: Supplementary file 1 [file DataSheet_1.zip › supplementary material/OSTEOPOROSIS-CIM/GCST90274799.tsv.gz_leave_one_out.pdf]

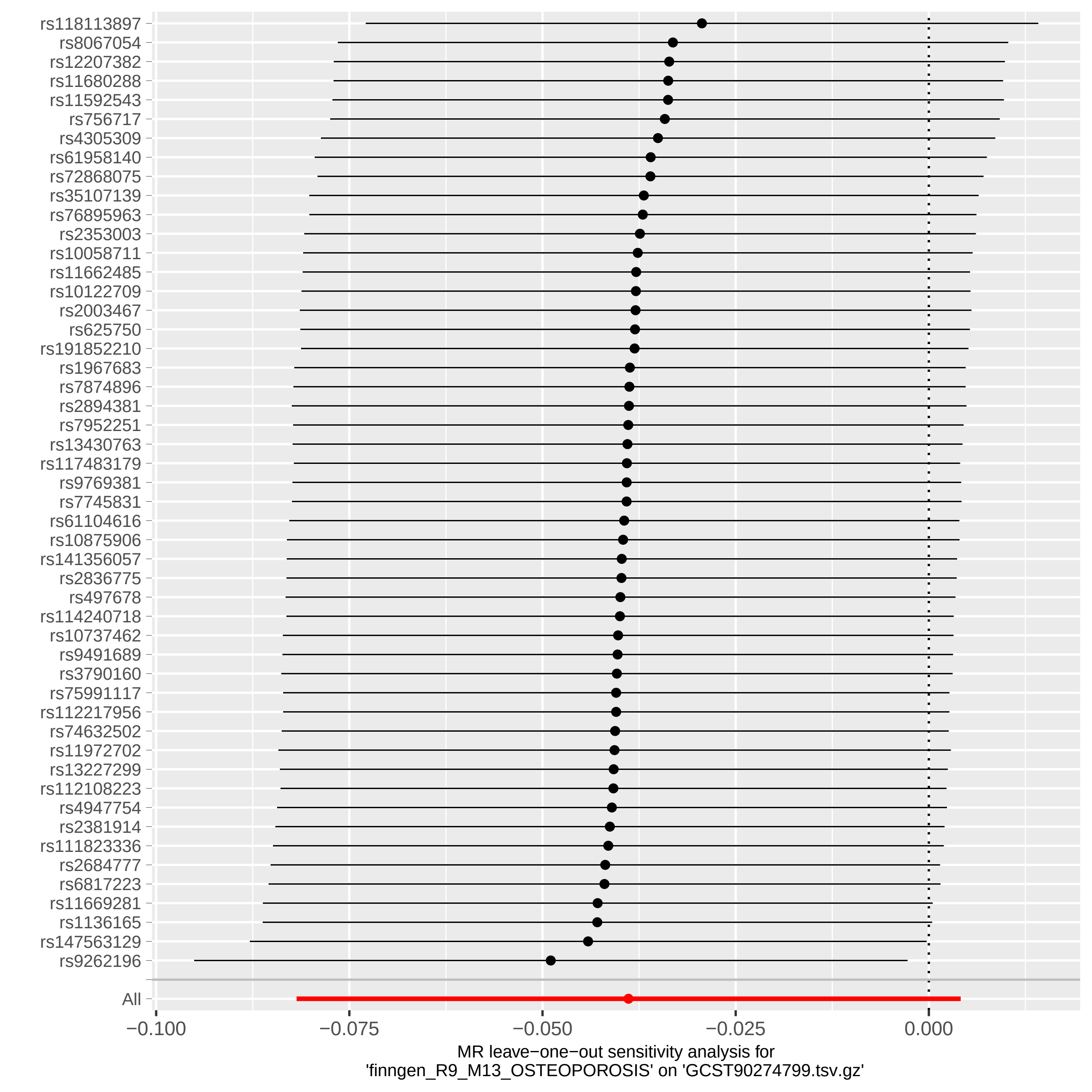
rs118113897
rs8067054
rs12207382
rs11680288
rs11592543
rs756717
rs4305309
rs61958140
rs72868075
rs35107139
rs76895963
rs2353003
rs10058711
rs11662485
rs10122709
rs2003467
rs625750
rs191852210
rs1967683
rs7874896
rs2894381
rs7952251
rs13430763
rs117483179
rs9769381
rs7745831
rs61104616
rs10875906
rs141356057
rs2836775
rs497678
rs114240718
rs10737462
rs9491689
rs3790160
rs75991117
rs112217956
rs74632502
rs11972702
rs13227299
rs112108223
rs4947754
rs2381914
rs111823336
rs2684777
rs6817223
rs11669281
rs1136165
rs147563129
rs9262196
All
−0.100
−0.075
−0.050
−0.025
0.000
MR leave−one−out sensitivity analysis for
'finngen_R9_M13_OSTEOPOROSIS' on 'GCST90274799.tsv.gz'
